# Recurrent ischemic stroke post-thrombolysis in an older Ghanaian woman

**DOI:** 10.4314/gmj.v58i2.9

**Published:** 2024-06

**Authors:** Kwadwo F Gyan, Priscilla A Opare-Addo, Moses Siaw-Frimpong, Kwasi Ankomah, Fred S Sarfo

**Affiliations:** 1 Komfo Anokye Teaching Hospital, Kumasi, Ghana; 2 Kwame Nkrumah University of Science & Technology, Kumasi, Ghana

**Keywords:** recurrent stroke, thrombolysis, early neurological deterioration

## Abstract

Acute ischemic stroke management has evolved through several paradigms. Currently, thrombolysis is recommended for patients who present within 4.5 hours of acute ischemic stroke. Early neurological deterioration post-thrombolysis, however, may occur through several mechanisms. We report a case of a 66-year-old Ghanaian woman with multiple co-morbidities who presented with sudden onset right-sided weakness and aphasia of 2 hours duration. A diagnosis of acute ischemic stroke was made based on clinical examination and a computerised tomography scan of the brain. She underwent successful thrombolysis with recovery of full neurological function. She, however, developed a second ischemic stroke within 72 hours, with the likely aetiology being large vessel occlusion. This was subsequently managed conservatively. Extensive evaluation and control of specific stroke aetiologies is required for the prevention of stroke recurrence post thrombolysis. Also, the establishment of comprehensive stroke centres which provide neurovascular interventions in sub-Saharan Africa can help reduce stroke mortality and morbidity in eligible patients.

**Funding:**

None declared

## Introduction

Acute stroke is the second leading cause of mortality and morbidity worldwide, with a global annual incidence of 12.2 million, representing a 70% increase from 1990 to 2019.^1^ There is a wide disparity in case fatality rate of stroke victims in sub-Saharan Africa (SSA) compared to the Western world. This is mainly attributable to weak healthcare systems.^2^ Use of intravenous thrombolytic therapy within 4.5 hours of symptom onset is currently recommended for acute ischemic stroke management.^3^

In SSA and low- to middle-income countries (LMICs), including Ghana, barriers such as cost, lack of expertise and resources, poor transportation and access to health facilities lead to missed timelines, sociocultural and religious practices and a lack of political will and health policy, negatively impact the implementation of acute stroke interventions.^4^,^5^ The uptake of thrombolytic therapy is only 3% in low-income countries, 19% in LMICs compared to 50% in high-income countries.^6^ However, Some centres in these resource-limited settings have emerged to implement thrombolysis.^7^,^8^ Emerging centres should put in place appropriate protocols for successful thrombolytic therapy implementation^8^ and also acknowledge the potential risks and possible outcomes of thrombolysis interventions, including early neurological recovery, early neurological deterioration(END), symptomatic and asymptomatic intracranial haemorrhage, extracranial haemorrhage and death.^9^ END occurs in 10% to 29.7% of patients post-thrombolysis through several mechanisms.^10^-^12^ We present a case report of an elderly Ghanaian woman who was successfully thrombolysed for an acute ischemic stroke but had recurrent ischemic stroke within 72 hours. We discuss the mechanisms for early neurological deterioration after thrombolysis for an acute ischemic stroke.

## Case Presentation

Our patient was a 66-year-old Ghanaian woman who had returned from the United States (U.S) for a family visit 2 weeks prior. She was a known diabetic and hypertensive for over 15 years, with coronary artery disease (had drugeluting stenting done 3 years prior to presentation), and not fully compliant on her medications. She was in this state of health until 2 hours prior to the presentation when she was noticed to be suddenly falling towards her right side while sitting down in a chair and having slurred speech with associated left-sided facial deviation. She could not move the right upper or lower limb and subsequently could not speak. There was no loss of consciousness, seizure, headache, neck stiffness, preceding fever, diaphoresis, chest pain, palpitations, vomiting, or breathlessness. She had no prior history of stroke, decompensated heart failure, arrhythmia, retroviral infection, hepatitis B or C, chronic kidney disease or malignancy. There was a family history of diabetes mellitus (DM) and hypertension but no family history of stroke, myocardial infarction or sudden cardiac death. She is a nurse, lives in the United States with her husband and children and does not drink alcohol or smoke cigarettes. The patient did not use herbal medications or any alternative medical solutions.

At presentation, an elderly woman of average build who was not pale, anicteric, afebrile, well hydrated, with no pedal oedema was examined. Her admission vitals were:

- Blood pressure- 169/110 mmHg
- Pulse rate- 78 beats/min
- Respiratory rate- 22 cycles/min
- Oxygen saturation- 98% on room air
- Temperature- 36.5 °C
- Random blood glucose- 8.7 mmol/L

Neurological examination showed that the patient was conscious but aphasic, with right upper motor neuron facial nerve palsy. The Medical Research Council (MRC) power grade was 1/5 in the right upper and lower limb, respectively. Deep tendon reflexes were brisk on the ipsilateral side with extensor plantar response. The National Institute of Health Stroke Scale (NIHSS) score was 20, and the Modified Rankin Score (MRS) of 5.

Her pulse was regular, of good volume, and non-collapsing with a palpable arterial wall. Jugular venous pressure was not raised, and her apex beat was not displaced. First and second heart sounds were present and normal with no added sounds. Carotid bruits were heard bilaterally. Chest and abdominal examinations were unremarkable.

**Figure 1:**
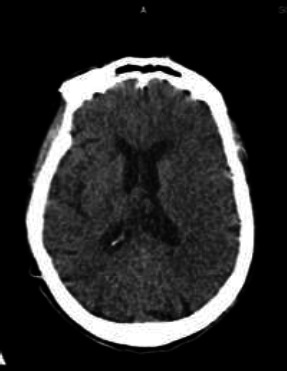
Plain CT brain showing no haemorrhage or acutely demarcated infarct

An urgent computed tomography (CT) scan of the brain showed no evidence of haemorrhage or acutely demarcated infarct **[Figure 1]**. However, there were hyperacute parenchymal changes with loss of grey-white matter differentiation on the left lentiform nucleus and insular ribbon.

### Other investigations

Electrocardiogram showed sinus rhythm with incomplete left bundle branch block and left ventricular hypertrophy. Carotid Doppler ultrasonography showed near occlusion of the right carotid bulb and right internal carotid artery, a plaque in the left internal carotid artery causing 44% stenosis and bilateral intimal wall thickening likely due to atherosclerotic changes.

Bedside trans-thoracic echocardiography showed biatrial enlargement with moderate concentric left ventricular hypertrophy and calcified aortic valves with mild insufficiency. The ejection fraction was approximately 50%.

Her urine dipstick was unremarkable. The complete blood count, renal function test, electrolytes, and liver function test results were within normal ranges. Glycated haemoglobin was 10.4%, suggestive of poor glycaemic control, and the lipid profile showed hypercholesterolemia.

She was immediately prepared for thrombolysis. Time between onset of symptoms and thrombolysis was approximately 3 hours. One hour into the administration of intravenous tissue plasminogen activator (t-PA) at 0.9 mg/kg given as 10% bolus, 90% continuous infusion over 60 minutes, patient was noticed to be moving the right side as well as speaking coherently.

She was subsequently admitted to the intensive care unit (ICU) for continuous monitoring and serial neurological assessment and was found to have no neurological or functional deficit after 48 hours. Her neurological (NIHSS) and functional (MRS) outcomes post thrombolysis at 2 hours, 24 hours and 48 hours were 2 and 1, 0 and 0, and 0 and 0, respectively. She was initiated on Tab Aspirin 150mg daily, Tab Atorvastatin 80mg nocte, Tab Citicholine 1g daily and continued on her antihypertensive medications (Tab Amlodipine 10mg daily, Tab Clonidine 0.1mg daily, Tab Triamterene/Hydrochlorothiazide 37.5/12.5mg daily, Tab Carvedilol 12.5mg 12hourly) and DM medications (Tab Dapagliflozin 10mg daily, Insulin degludec 30units daily).

At this time, carotid endarterectomy (CEA) or carotid artery stenting (CAS) was recommended within 14 days to reduce her risk of recurrent, contralateral or fatal stroke.^13^, ^14^ Since the intervention could not be done at our facility, the family planned expedited return to the U.S due to her insurance coverage for the procedure there.

**Figure 2:**
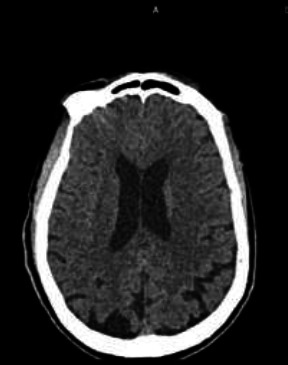
A Plain CT brain done on recurrence showing no haemorrhage or acutely demarcated infarct

However, three days post-thrombolysis, the patient was noticed to have developed sudden onset right-sided weakness with associated inability to speak again. There was no loss of consciousness or seizure, no fever, cough or breathlessness, no hematemesis, haemoptysis, haematuria, hematochezia, melena or evidence of bleeding on any mucosal surface, skin or joint. Neurological examination revealed she was conscious but aphasic, with right upper motor neuron facial nerve palsy. The MRC power grade was 3/5 in the right upper limb and 1/5 in the right lower limb. Deep tendon reflexes were brisk on the ipsilateral side with extensor plantar response. NIHSS score was 19, and MRS was 5. CT scan of the brain on recurrence of hemiparesis showed no haemorrhage or acutely demarcated infarct (Figure 2)

**Figure 3:**
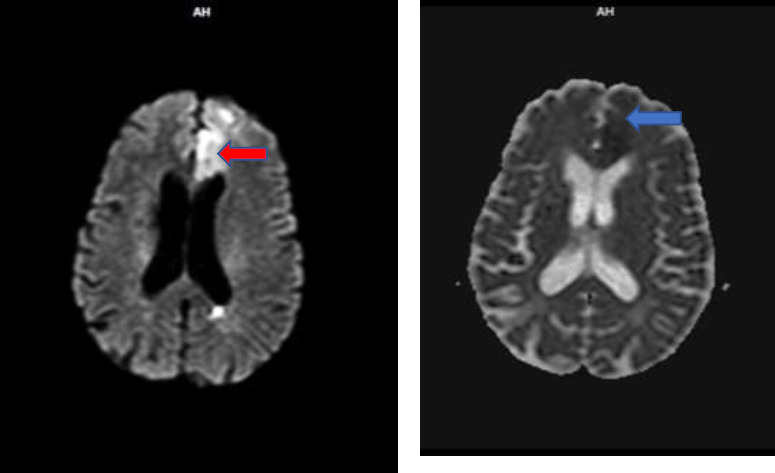
Magnetic resonance imaging (MRI) showing 4.5 x 1.8 cm wedged-shaped cortical -a subcortical region in the left frontal lobe, which is hyperintense (red arrow) on diffusion-weighted imaging (DWI) and shows subtle hypointensity (blue arrow) on apparent diffusion coefficient (ADC) sequence

However, magnetic resonance imaging (MRI) showed features suggestive of hyperacute left anterior cerebral artery territorial infarct; bilateral, symmetrical, periventricular T2/STIR hyperintensities extending to the deep white matter (Fazekas 2) (Figure 3)

She was subsequently managed conservatively on the previously prescribed medications for secondary prevention as (repeat) thrombolysis is contraindicated within 3 months of ischemic stroke.^3^,^15^ Also, she was considered not to benefit from CEA as the ipsilateral symptomatic carotid stenosis was mild while the contralateral asymptomatic stenosis was rather severe, and she was acutely experiencing a major devastating stroke.^13^ Speech therapy and physiotherapy were initiated, and she was discharged on admission day 11 with stable vital signs, optimised blood pressure and glucose control. She was conscious but aphasic, and her MRS was 5. She was transferred to the U.S., where she originally resided with her family, for the continuation of medical care and for social support. She is currently receiving care at a stroke rehabilitation centre, and her MRS remains 5.

### Consent

Written/verbal informed consent was obtained from the patient and her next of kin to publish this report under the journal's patient consent policy.

## Discussion

Post thrombolysis, early neurological deterioration (END), which is usually defined as a drop of 4 points or more on the NIHSS within the first 48 to 72 hours after acute stroke, occurs in about 10% to 29.8% of patients.^10^-^12^ It is attributable to causes such as worsening of index stroke, malignant oedema, early post-stroke seizures, symptomatic intracranial haemorrhage, recurrent ischemic stroke or unexplained.^10^,^11^ Marie Tisserand et al demonstrated significant extra-penumbral infarct growth in most patients with unexplained END.^11^ Recurrent ischemic stroke, defined as the presentation of newly developed neurological deficits or exacerbation of initial neurological deficits in the previously re-canalized vessel or another vascular territory that was not initially involved, due to newly developed occlusion in major extracranial or intracranial arteries based on CT angiography or magnetic resonance imaging(MRI) studies occurs in 2.6% of patients post-thrombolysis and is significantly associated with atrial fibrillation.^10^,^16^ Our patient, however, did not have atrial fibrillation.

Mechanisms that lead to recurrent ischemic stroke include arterial re-occlusion, proximal extension of the thrombus, distal embolisation of thrombus or fragment embolisation, and cardioembolism from disintegration of pre-existing intra-cardiac or valvular thrombus.^16^-^18^ Arterial re-occlusion accounts for some cases of END, but the clinical scenario, as seen in our patient, is more often deterioration after initial improvement rather than END alone.^17^,^18^ Fragment embolisation or proximal extension of the thrombus may also be a plausible explanation of her presentation. Ultrasonography is limited in sensitivity and specificity compared with MRI, which has a high resolution and sensitivity for identifying vulnerable plaques.^19^ MRI study of the carotids as well as MRA of the cerebral vascular territories was, however, not done to be able to confirm this as the cause of the stroke. Although trans-thoracic echocardiography did not show preexisting intra-cardiac or valvular thrombus, cardioembolism cannot be ruled out as she had evidence of ischemic heart disease. Our patient has several risk factors for ischemic stroke and its recurrence, including DM, hypertension, atherosclerosis, dyslipidaemia and recent ischemic stroke.

Protocols encompass measures to help avert END vis-a-vis inclusion and exclusion criteria, pre and post-procedure investigations, and monitoring, among others. 3,15 Although routine non-contrast head CT at 24 hours postthrombolysis to evaluate for haemorrhage prior to the initiation of antiplatelet therapy is recommended, it was not done. According to the Follow-up Imaging After Thrombolysis-FIAT trial, routine CT had no added benefit in the absence of clinical decompensation.^20^ Some negative outcomes are unpredictable and potentially unpreventable as our patient developed stroke recurrence after 72 hours when efforts were being made to transfer for possible CEA or CAS to reduce her risk of recurrent, contralateral or fatal stroke.

## Conclusion

Early Recurrence of stroke can occur even after successful thrombolysis. A thorough evaluation of the underlying cause and risk factors is necessary for secondary stroke prevention after thrombolysis. Capacity building and infrastructure is urgently required for the establishment of comprehensive stroke centres capable for performing current cutting-edge interventions in acute stroke management to reduce stroke mortality and morbidity in SSA countries like Ghana.
